# Crania Americana; or a Comparative View of the Skulls of Various Aboriginal Nations of North and South America; to Which Is Prefixed an Essay on the Varieties of the Human Species

**Published:** 1840-08

**Authors:** 


### REVIEWS.

---

Art. V.—*Crania Americana; or a comparative view of the Skulls of various Aboriginal nations of North and South America: To which is prefixed an Essay on the Varieties of the Human Species. Illustrated by seventy-eight plates, and a colored map.* By Samuel George Morton, M.D., Professor of Anatomy in the Medical Department of Pennsylvania College at Philadelphia; member of the Academy of Natural Sciences of Philadelphia; of the American Philosophical Society; of the Historical Society of Pennsylvania; of the Boston Society of Natural History, &c., &c., Philadelphia; J. Dobson, Chesnut street. London; Lumpkin, Marshall & Co., 1839. Letter press, p. 296, folio.

In the last number of the Journal a promise was given, that, on renewing our remarks on this distinguished work, which, though the production of an individual, might well be called national, we should endeavour to enrich our notice of it, with a greater amount of the special and detailed information which it contains, than was introduced into our former article respecting it. And our promise must be redeemed. In effecting this redemption however, it is not our intention to deal exclusively in specialties and details. We shall not lose sight of those great principles of science, nor fail occasionally to refer to them, which constitute an essential element of liberal knowledge, and without which *science* would deservedly forfeit its name and character, and degenerate into disjointed and common-place intelligence.

In a special manner we shall not fail to bear in mind that fundamental and all-controlling truth in physiology, whose influence and value have been heretofore too little attended to, and too lightly appreciated, especially by anthropologists—that the *nervous* is the *master-tissue* of living organized matter, and that that portion of it called *the brain* is chiefly instrumental in creating distinctions between human individuals, as well as between varieties and races of men; and in giving to some of them a decided and permanent superiority over others. For, as intimated on a former occasion, that truth, when skilfully and ably employed, is calculated, far beyond any other, to shed light on the history and philosophy of man. Nor is its influence limited to the past and the present. To a certain extent it is also instinct with a spirit of foresight, and fitted to lift the curtain suspended in most cases between mankind and the future, and disclose to them somewhat of the destiny that awaits them.

Our reason for entertaining this opinion, which we know will not be likely to be favoured, at first view, with the belief of the *multitude*, may be succinctly stated. We can foresee what man would be, were he converted, by a well-conducted and thorough course of education and discipline, into a truly *rational* and *moral* being—were all his *higher* and *nobler faculties* we mean, so strengthened and trained, as to have the complete control of his inferior faculties, and his will so predominant as to have a corresponding mastery over *them.* And we can foresee what the condition of the world would be, were it peopled with beings so elevated and excellent. But were time allowed us, and were the discussion a suitable one for the present occasion, it would be easy to show, that a complete knowledge of the brain and nerves, and of the best scheme of exercising and improving them, would enable mankind to produce in the world the state and condition of things, to which we have alluded. And that such knowledge and scheme, in no very limited degree, will be, in time to come, one of tne products of phrenology, is highly probable. The future then, under such circumstances, is in part at least revealed to them. Though they have not obtained possession of the land of promise, they enjoy an antepast of it in prospect from the Pisgah they have ascended.

Such a view of things cannot, we think, be regarded as either fanciful or extravagant. Far from it. Founded on acknowledged truth, and constructed of sound materials, it cannot be otherwise than substantial and lasting. But we must proceed, without further preface, to our task of analysis.

In our article contained in the preceding number of this Journal, it was observed that Dr. Morton commences his work with an "Essay on the Varieties of the Human Species." And, for its extent, it is decidedly the most interesting and instructive we have ever perused. It contains the result of much and varied reading and research; its matter, though compressed within the narrowest limits, is rendered perfectly intelligible, by the simplicity, plainness, and perspicuity of the style; and, added to its historical information, it is interspersed with many striking and valuable remarks.

Alluding to the extensive, if not *universal* population of the earth, at the earliest epoch known to history, the writer correctly states, that "the *oldest records* seldom allude to an *uninhabited* country." Yet do some of those "records" go back to within a few centuries of the flood, and still find populous, powerful, and even cultivated nations. Indeed, according to our most accredited systems of chronology, within a single century and a year after the subsidence of the deluge, the east was sufficiently populous, and the inhabitants sufficiently versed in the mechanical arts to erect the tower of Babel—a building believed to be superior in magnitude, and vastly superior in grandeur of design, to any architectural monument subsequently attempted. In about twenty-six years afterwards the Chaldean monarchy was founded by Nimrod—and in fourteen years more the Chinese monarchy, by some of the master spirits of the time. These facts bespeak an increase of the human family, at that period, far surpassing in rapidity any thing the world exhibits at present.

Dr. Morton farther states, with equal correctness, and as an evidence of great *antiquity* of residence, that so strong is the attachment of each variety and people to their own dwelling places, that they greatly prefer them to all others. "Thus, says he the Eskimaux, surrounded by an atmosphere that *freezes mercury*, rejoices in his snowy deserts, and has pined in unhappiness when removed to more genial climes. On the other hand, the native of the torrid regions of Africa, oppressed by a vertical sun, and delirious with thirst, thinks no part of the world so desirable and delightful as his own." So truly might the poet have sung of the predilection for home inherent in the inhabitants of every region, whether hot or cold, parched or humid, as he did of that which he represented to be so vivid and glowing in those who are born and reared amid polar snows, and immediately beneath the path of the sun.

"The shuddering tenant of the frigid zone,
"Boldly proclaims the *happiest home* his own,
"Extols the treasures of his stormy seas,
"And his long nights of revelry and ease;
"The naked negro, panting at the line,
"Boasts of his golden sands, and palmy wine;
"Basks in the glare, or stems the tepid wave,
"And thanks his gods for all the good they gave."

Again says our author; "From remote ages" (he might have said the *most remote*) "the inhabitants of every extended locality have been marked by certain physical and moral peculiarities, common among themselves, and serving to distinguish them from all other people. The Arabians are at this time precisely what they were in the days of the patriarchs; the Hindoos have altered in nothing, since they were described by the earliest writers; nor have three thousand years" (he might have extended the period to three thousand *five hundred*) "made any difference in the skin and hair (and he might have added *features*) of the Negro. In like manner the characteristic features of the Jews may be recognized in the sculpture of the temples of Laxor and Karrak, in Egypt, where they have been depicted for nearly thirty centuries."

Throughout this whole passage, the writer has restrained himself not a little *within* the limits to which he might in verity have carried his account of the stubborn permanency of the human complexion, features, and figure. Documents not to be controverted can be adduced to show, that *six-and-thirty hundred* years ago, the Jews in Egypt bore, in every characteristic trait of person and countenance, the most striking and precise resemblance to the Jews of the present day. Neither their residence in Palestine, nor their subsequent banishment and dispersion into every climate, situation, and country, has produced in either their personal figure, the form of their head, the colour of their skin, or the lineaments of their countenance any well-marked or even perceptible change. All these facts, and many others that might be easily detailed, speak to the same effect. And they testify conclusively to the immense antiquity of the crowded population of the earth, and the absolute impossibility of specifying any

*natural* causes, physical, moral, or intellectual, capable of producing the numerous, immense, and dissimilar alterations in man, requisite to change and diversify him from *unity of origin* into the now-existing and strongly-marked varieties and races. And all attempts to that effect have not only failed to do good, by the discovery of truth in history or science, but have done positive mischief, by giving a wider scope and deeper inveteracy to error and prejudice. For the sake of religion therefore, as well as morality, unsophisticated reason, and sound philosophy, researches of the kind should be discountenanced and abandoned. No natural causes now in existence have the slightest tendency to change one race of men into another—or even *one variety of the same race into another*. All efforts made to propagate hypothesis in opposition to this, do violence to truth, and give countenance to error—and are therefore necessarily of pernicious tendency. For error of every description is a positive and never-failing source of evil. Nor are these remarks designed to have the slightest bearing on the doctrine of the *original unity of man*. They relate exclusively to the *causes of his diversification*. And we reiterate our thorough conviction, that nothing short of the POWER which created him could have produced the change.

To render his readers the more familiar with the climates and localities of the different races and varieties of man, Dr. Morton has prefixed to his work a well prepared map of the earth, indicating by different shades of colour, the places of dwelling of the several races.

The effect of an examination of this map is singular, and exhibits in the most striking and forcible light the vast superiority of the Caucasian race in every thing but numbers—

their superiority especially in science, literature, and the arts, power and government; and in every thing else subservient to the comforts of peace, the operations of war, and the elegance and elevation of condition and standing.

The Caucasians are in all respects the masters of the world, though they do not we believe constitute a fifth part of its inhabitants, nor cover perhaps more than one-eighth or tenth part of its surface. It is curious, as well as instructive, in a special manner, to compare the diminutive size of Great Britain, with the measureless dimensions of the nations and territories she has conquered and sways. She occupies on the map we have referred to but little more than a mere *speck* of space, which those who know not its position have difficulty in finding; while her fleets cover every sea and ocean, her arms are almost uniformly and every where triumphant, and her power is felt in every corner of the peopled globe. Nor can even the inferior animals in the north, the tropics, or the south, and whether they wing the air, cleave the waters, or move on solid ground, escape either by flight, concealment, or resistance, the devices of her artfulness, the snares of her hand, or the unlimited sweep and mightiness of her arm. And what is the source of this power and influence? We reply unhesitatingly the functions of the brain—of the largest, best developed, and best conditioned brain belonging to man. And if this brain be accompanied by bodies of the best size and shape, and the most adroit and vigorous in action, let it not be forgotten that brain and nerves, being the master tissue of the system, have no little concern as well in the production of those excellencies of quality and endowment, in other portions of the body, as in their superintendence, maintenance, and regulation when produced. For that the brain, when of the highest order and in the best condition, imparts to the other tissues and organs of the body, somewhat of the tone and character of its own distinguished qualities, is as certain as that moisture and sunlight, warmth and atmospherical air, co-operating with each other in a well-adjusted union, contribute to the growth and excellence of vegetables.

In a word, Great Britain is peopled chiefly by Anglo-Saxons, the most highly endowed variety of the Caucasian race. Their brains are superior in size, and more perfect in figure than the brains of any other variety; and, from temperament and exercise, they are in the best condition. In function therefore they are the *most powerful* at least, if not the most *active.* And hence the surpassing strength and grandeur at home, and the influence and sway over the other nations of the earth, of those who possess them. The vast and astonishing productions of art in Great Britain, her boundless resources of comfort and enjoyment in peace, and her unequalled means of defence and annoyance in war, are as literally the growth of the brains of her inhabitants, as her oaks, and elms, and ash-trees are of her soil. We shall only add, that the inhabitants of the United States, being also of the best Caucasian stock, and youthful, elastic, and vigorous, as a nation, and enjoying the influence of other circumstances as favorable to the production and perfection of mental and corporeal excellencies as nature can frame, or imagination conceive — in the midst and under the immediate agency of such advantages, the people of the United States promise to be even more than the Britons of future ages.

Having finished his history and geography of the other races of men, our author commences his exposition of the American race. And in this enterprise lie, as was designed to be the case, the chief interest and value of his book.

In the beginning of this exposition he very properly notices a phenomenon which, if not peculiar to the American race, is more strongly marked in them, than in any other people. It is the striking similarity, both physical and moral, very especially in complexion, countenance, and hair, which attaches to them *as a whole.* True; they have their varieties; tribes and communities of them permanently differing in sundry respects from each other. But the differences are not so prominent as those which characterize the varieties of other races. They are much less striking, for example, than the differences which exist betwen real Negroes, Caffres, Hottentots, and Boschesemen—between Arabs, Persians, Hindoos, and Circassians—or even between Germans, Italians and Irish.

When a nation composed of the same people is of great extent from north to south, as is the case with China and Hindostan, Arabia and the United States, the inhabitants of the two extremes differ very percepibly from each other in complexion and figure. The natives of the North are comparatively fair and ruddy, full in flesh, broad in frame, and bulky in proportion to their height. Of the natives of the South, the reverse is true. They are darker in complexion, rather taller, slenderer in frame, and less muscular and weighty in person. This truth is illustrated and confirmed by the appearance of the House of Representatives of the Congress of the United States. When the House is full, a practised eye can readily discriminate, by their complexion and figure, between the members from the northern, middle, and southern States.

Among the aborigines of our country, this phenomenon, so uniform elsewhere, does not present itself—at least not so strikingly or to the same extent. Of the American continent the extent from north to south is considerably greater, than that of the three continents of the Old World—the southern extreme of the New World being many degrees to the south of the Cape of Good Hope. To this amount of latitude add the influence of its mountains and high table-land, broad plains and prairies, and deep and extensive vallies, and the New World possesses necessarily a greater diversity of climate than the Old. From this unequalled diversity of climate and situation at least, if not also of other physical causes, we might well feel authorized to expect a correspondingly greater diversity of complexion and countenance among its aboriginal inhabitants. But the reverse of this is true. The complexional diversity is greatly inferior. From the confines of Labrador to the heights of Cape Horne, and from the Atlantic to the Pacific ocean, the complexion of the Indian is nearly the same. So is the general physiognomy, with the whole physical appearance and character; and so in a high degree are the moral qualities. Neither latitude nor elevation of dwelling; the freedom and purity of mountain air; the fogs and stagnant atmosphere of vallies; the deep shades of primeval forests; nor the unmitigated and unaverted blaze of sunbeams descending on prairies, produce on the aspect of the roving and houseless native, any very strongly marked, or permanent effect. In the north and south, as well as in the torrid belt of the Equator, and on the mountain, the hill, and the plain, the deep valley, and the interminable prairie, his complexion, figure, and features remain nearly the same. Or if any material difference exist, it is found only among the natives of Mexico and Peru. More singular still. So little influence has latitude and temperature on the complexion of the American Indian, that it is, in many instances, darker in tribes inhabiting cold and temperate regions, and residing on cool and breezy mountains, than it is in the inhabitants of the ill-ventilated and sultry vallies at their feet, and of the still more burning regions of the Torrid Zone. The well known fact, that amidst the scorching heats of equatorial America, there are found neither real Negroes, nor any thing resembling them, while that race abound in the much milder climates of Oceanica, and elsewhere without the Tropics—that fact alone affords ample and conclusive proof, that *mere temperature*, whether high or low, and whether it be accompanied by moisture or dryness, takes no part in the production of the Ethiopian complexion or hair, features or figure. Indeed the entire history of the aborigines of America, is a sound and overwhelming body of evidence in refutation of the *temperature and climate hypothesis*, which is but the ricketty offspring of ignorance and prejudice; and the nurseling of error, superstition and obstinacy.

Notwithstanding however the similitude, in complexion, of the different varieties and tribes of the American race, the differences in the size and configuration of their heads is sufficiently striking. And in the judicious and accurate exposition of that topic, consists the highest excellence of the work we are examining. Of the differences in the form, though most of them are natural, yet not a few are the product of art. The latter are effected by pressure applied to the heads of children, from birth, until the skull is sufficiently ossified, to retain the flattened or elongated form it has received—for either form is given, according to the taste of the nation or tribe. Those Indians whose crania are most odiously disfigured by this process, are the Charibs, the Natchez, a variety of the race believed to be extinct, the ancient Peruvians, those whose skulls are extracted from the ancient tumuli of the country, and sundry tribes resident on the Columbia river, and scattered along the shores of the Pacific ocean. Most of the tribes and nations between the base of the Rocky Mountains and the Atlantic ocean, allow their heads to retain, unmolested, the form that nature gives them. It is worthy of remark, that in whatever degree the shape of the skull may be changed by pressure, its size is but little if at all affected by it. The cavities of the most deformed skulls are found to be as capacious, as the cavities of those allowed to preserve their natural figures.

Nor has it been indisputably ascertained that either intellect or health, strenth or longevity, or any other important attribute of mind or body, is materially injured by the pressure and disfiguration of the brain. Completely to solve this problem, however, additional observation and research are essential—observation and research moreover especially directed to the points in question—a measure which we believe has not yet been pursued. As far as our own *suspicion* may avail, we deem it hardly possible that pressure so severe and long-continued as greatly and permanently to derange the shape of the brain, can be practised with impunity. We can hardly doubt, that most feeble children are destroyed by the process; and that the more robust and vigorous ones only survive it. Those tribes and nations, moreover, who now practise it, are in general less elegantly proportioned in their limbs, less masculine and powerful in their persons, and less expert and formidable in war, than those who decline it, and suffer their heads to take their natural shape. Hence the Indians on the west side of the Rocky Mountains are inferior in strength and bodily activity, less warlike, and less dangerous to hunters, trappers, and traders, than those on the east side of them. The real causes of these differences however are yet to be disclosed. They *may* be the presure and disfiguration of the brain alone; they may embrace that combined with other agencies—or it is even *possible* that the compression may have no influence in the matter. As relates to the process of giving an artificial shape to the head, we shall only add, that Dr. Morton possesses a model of the machine, by which it is effected; and that he has inserted a description, accompanied by an accurate engraving of it in his book. There exists therefore no longer any doubt of the *reality* of the process; whereas, with not a few persons, such doubt, until recently, *did* exist. We speak from *experience;* because, by ourselves, the point has been seriously questioned. And should we even be told that it has been pronounced by us "a mistake"—"a fiction"—and "a traveller's tale"—we should be compelled perhaps, in honesty, to plead "guilty" to the charge. It is worthy of remark, that the altered form of the head is not *hereditary*, but terminates with the individual, and is, in every case, an artificial production. Every infant, therefore, in tribes where the disfiguration is fashionable, is compelled to submit to the torturing process. Such at least is our author's opinion on the subject. From this, if true, the inference may be safely drawn, that flat-headedness does not become *constitutional;* because every constitutional quality is known to be transmissible from parents to their offspring.

Before entering on the representation and formal exposition of their "Crania," our author offers some interesting remarks on the *Stature* of the American race.

"That race," to use his own language, "presents some remarkable contrasts. The Patagonians" (of which the Araucanians are the most distinguished branch) "are the tallest nation on the American continent. Of five hundred of them seen together by Commodore Byron, the shortest were at least *four inches* taller than his own men. Capt. Wallace took the pains to measure many of them, among whom one was six feet seven, and several six feet five; but the greater part of them were from five feet ten, to six feet. On the other hand, Humboldt found the Chaymas, and some other tribes of the upper Orinoco to be remarkably short. The Pourys and Coroados of Brazil are diminutive races, while the Albipones of Paraguay are, to a man of gigantic proportions. The Muscogee (Creeks) are strikingly tall and athletic—a full size larger than the Europeans. Many nations also both of North and South America are remarkable for the perfect symmetry of their persons." In these, and other facts, as the writer very justly observes, " there is ample evidence to disprove the hypothesis of some closet naturalists, that the physical man of the New World is of defective and degenerate organization."

In farther testimony of this latter truth, it might be added, that, be the cause what it may, the Anglo-Saxon stock of the Caucasian race acquire in America (especially in the United States) a loftier stature than they possess in Great Britain. Hence the American sons of English and Irishmen are usually taller than their sires; and the military step is ordered to be a little longer in the former than in the latter country. Give to the Aborigines of the New World, then, a nervous system in perfect equality, as regards excellence in size, form, temperament, and condition, with the other portions of their organization, and they will rival at least, if they do not surpass the leading variety of the Caucasian race. But in brain, by which alone elevated and efficient standing and character can be attained and preserved, they are deficient. And hence their inferiority. But we must proceed in our analysis.

Those elements of Dr. Morton's labours, from which can be formed the most just and accurate view of the drift and usefulness of the work, are his numerous delineations and admeasurements of skulls. In his delineations he represents the differences of those skulls *in form*, and in his admeasurements their difference *in size*, with a correctness and exactitude neither equalled nor even aimed at, as far as our information reaches, by any other writer. Of the interest and value of his delineations no satisfactory knowledge can be conveyed by us to the reader, except by a lithograph or an engraved presentation of his plates, which cannot, of course, be given in this article. That information can be acquired only by an examination of his book. From a brief account however of the details of his admeasurements, some conception can be formed of their utility and importance. And that account shall be offered in the writer's own words.

"*Anatomical Measurements.*

"These measurements are derived from one hundred and forty-seven skulls of American Indians, of forty different nations and tribes; and the crania are all of adult persons, and unaltered by art. * * * It is necessary to premise the manner in which the measurements have been taken.

"The *longitudinal diameter*" (of the skull) "is measured from the most prominent part of the os frontis, between the superciliary ridges, to the extreme end of the occiput.

"The *parietal diameter* is measured between the most distant points of the parietal bones, which are, for the most part, the protuberances of these bones.

"The *frontal diameter* is taken between the anterior inferior angles of the parietal bones.

"The *vertical diameter* is measured from the fossa between the condyles of the occipital bones to the top of the skull.

"The *inter-mastoid arch* is measured, with a graduated tape, from the point of one mastoid process to the other, over the external table of the skull.

"The *inter-mastoid line* is the distance, in a straight line, between the points of the mastoid processes.

"The *occipito-frontal arch* is measured by a tape over the surface of the cranium, from the posterior margin of the foramen magnum, to the suture which connects the os frontis with the bones of the nose.

"The *horizontal periphery* is measured by passing a tape around the cranium, so as to touch the os frontis immediately above the superciliary ridges, and the most prominent part of the occipital bone.

"The *length of the head and face* is measured from the margin of the upper jaw, to the most distant point of the occiput.

"The *zygomatic diameter* is the distance, in a right line, between the most prominent points of the zygoma.

"The *facial angle* is ascertained by an instrument of ingenious construction," &c.

The author's entire description of this instrument it would be useless to insert; because, unless it were accompanied by an engraving, it could not be understood. It must therefore give place to intelligible matter.

From this account of Dr. Morton's measurements, the osteologist will perceive their fitness to give the dimensions of the cranium to be sufficiently perfect for the purposes aimed at. As appears to us, they are wanting in nothing. And the execution of them we believe to have been equally unexceptionable. When to these again is added the measurement of the internal capacity of the skull, both as *a whole*, and as divided into its several regions, in the character of sub-cavities or chambers, every thing is done, as respects the ascertainment of dimension and figure, that can be desired or devised. The whole moreover is well calculated to supply the mental philosopher with the data necessary to enable him to calculate the amount, both proportional and abstract, of intellect, morals, and animality, and therefore of general character, belonging either to individual man, or to races of men, and their subordinate divisions. The real results and usefulness of the writer's measurements can be learnt and appreciated only from specifications of them. Some of them are as follows:

" PLATE V.—ANCIENT PERUVIAN.

" *Measurements.*

| | |
|---|---|
| " Longitudinal diameter, - - | 6.7 inches. |
| Parietal diameter, - - - | 4.5 " |
| Frontal diameter, - - - | 4.1 " |
| Vertical diameter, - - - | 4.1 " |
| Inter-mastoid arch, - - - | 11.5 " |
| Inter-mastoid line, - - - | 3.6 " |
| Occipito-frontal arch, - - - | 14.2 " |
| Horizontal periphery, - - - | 18 " |
| Extreme length of head and face, | 8.8 " |
| Internal capacity, - - - | 65.5 cubic inches. |
| Capacity of anterior chamber, - | 19.75 " " |
| Capacity of the posterior chamber, | 45.75 " " |
| Capacity of the coronal region, - | 12.75 " " |
| Facial angle, - - - - - | 61 degrees." |

This cranium, though small, may be received as a fair specimen of the average size of the ancient Peruvian head. Compared to the animal portion of the brain, the intellectual and moral portions are small. It is believed however that the shape of the skull is not natural, but has been altered by pressure.

" THE INCA PERUVIANS.

" PLATE IX.—PERUVIAN FROM THE TEMPLE OF THE SUN.

" A strikingly characteristic Peruvian head, for which I am indebted to Dr. Ruschemberger. As is common in this series of skulls, the parietal and longitudinal diameters are nearly the same.

"*Measurements.*

| | | |
|---|---|---|
| "Longitudinal diameter, - - | 6.1 | inches. |
| Parietal diameter, - - - | 6. | " |
| Frontal diameter, - - - | 4.7 | " |
| Vertical diameter, - - - | 5.5 | " |
| Inter-mastoid arch, - - - | 16. | " |
| Inter-mastoid line, - - - | 4.5 | " |
| Occipito-frontal arch, - - - | 14.1 | " |
| Horizontal periphery, - - - | 19.5 | " |
| Internal capacity, - - - | 83 | cubic inches. |
| Capacity of the anterior chamber, | 33.5 | " " |
| Capacity of the posterior chamber, | 49.5 | " " |
| Capacity of the coronal region, - | 15.75 | " " |
| Facial angle, - - - - | 81 | degrees." |

The reader will perceive that this Inca or modern Peruvian skull is much larger and better proportioned than the preceding ancient one. It is indicative therefore of having belonged to a superior stock of men—a stock well calculated to prove victors and conquerors, in warfare with the others. And such we doubt not they did prove. They invaded the ancient Peruvians, and banished, enslaved, or exterminated them—most probably the latter. Such, we feel persuaded, is always and necessarily the case, as regards the *permanent* conquest and overthrow of nations. The conquerors have the larger and better proportioned brains.

The reverse of this we apprehend is never true. In no instance does a people, with smaller and worse developed brains, achieve the *permanent* conquest of a people endowed with larger and better ones. True; the Moors, an inferior caste of the Caucasian race, conquered a large portion of Spain, and held possession of it for centuries, though peopled by Caucasians of a higher order. But they were ultimately conquered in turn, by their superiors in cerebral endowment, and banished from the country. Nor is it at all probable, that any event, in the revolutions of time, will ever recall them to Granada and the Alhambra.

" PLATE XVII.—MEXICAN.

" *Measurements.*

| | | |
|---|---|---|
| " Longitudinal diameter, - - | 6.8 | inches. |
| Parietal diameter, - - - | 5.5 | " |
| Frontal diameter, - - - | 4.6 | " |
| Vertical diameter, - - - | 6. | " |
| Inter-mastoid arch, - - - | 15.6 | " |
| Inter-mastoid line, - - - | 4.4 | " |
| Occipito-frontal arch, - - - | 14.6 | " |
| Horizontal periphery, - - - | 19.9 | " |
| Internal capacity, - - - | 89.5 | " |
| Capacity of anterior chamber, - | 33.5 | " |
| Capacity of posterior chamber, - | 56. | cubic inches. |
| Capacity of coronal region, - | 19.5 | " " |
| Facial angle, - - - - - | 80. | degrees." |

This head, superior to the Peruvian, is believed to be also above the *average* of the Mexican head.

" PLATE XXIII—SEMINOLE.

" *Measurements.*

| | | |
|---|---|---|
| " Longitudinal diameter, - - | 7.1 | inches. |
| Parietal diameter, - - - | 5.6 | " |
| Frontal diameter, - - - | 4.7 | " |
| Vertical diameter, - - - | 5.5 | " |
| Inter-mastoid arch, - - - | 15. | " |
| Inter-mastoid line, - - - | 4.1 | " |
| Occipito-frontal arch, - - - | 14.8 | " |
| Horizontal periphery, - - - | 20.3 | " |
| Internal capacity, - - - | 89. | cubic inches. |
| Capacity of the anterior chamber, | 52. | " " |

| | | |
|---|---|---|
| Capacity of the posterior chamber, - | 37½ | cubic inches. |
| Capacity of the coronal region, - | 19½ | " " |
| Facial angle, - - - - | 78 | degrees." |

This, being probably about an average standard of the national head is indicative of no common capacity, and shows the Seminoles to be a well endowed variety of the American race. No wonder that, in a country like Florida, abounding in countless and impenetrable places of defence, concealment and annoyance, the conquest of them should be difficult. In the capacities of the anterior and posterior chambers we suspect some mistake.

Without giving their measurements, we shall merely mention that the skulls of the Creeks and Cherokees are of the same order with those of the Seminoles. And their history testifies that those two nations also are warlike and formidable. And the heads of the Chippeways, another nation similar in character, are fully equal, if not superior. So are the heads of the Miamis and Potowatomies—nations that have been obstinate and destructive in war. Of the "five nations" the same is true. Their brains as well as the history of their wars, show them to possess a high station in the American race.

The Osage alone we believe excepted, the Indians west of the Mississippi and east of the Rocky Mountains, are of an inferior order. This appears from the following measurements, which may be regarded as giving about the average (or rather a little more) of the skulls of those nations. Some of them however are fearfully ferocious and sanguinary.

"PLATE XL.—BLACKFOOT.

"*Measurements.*

| | | |
|---|---|---|
| "Longitudinal diameter, - - | 7.1 | inches. |
| Parietal diameter, - - - | 5.4 | " |
| Frontal diameter, - - - | 4.3 | " |
| Vertical diameter, - - - | 5.1 | " |
| Inter-mastoid arch, - - - | 13.8 | " |
| Inter-mastoid line, - - - | 4.3 | " |
| Occipito-frontal arch, - - - | 14. | " |
| Horizontal periphery, - - - | 19.9 | " |
| Internal capacity, - - - | 77 | cubic inches. |
| Capacity of the anterior chamber, | 33½ | " " |
| Capacity of the posterior chamber, | 44½ | " " |
| Capacity of the coronal region, - | 18.2 | " " |
| Facial angle, - - - - - | 78 | degrees." |

"THE FLAT-HEAD TRIBES OF THE COLUMBIA RIVER."

In one respect these Indians may be pronounced an *anomaly;* yet the singular problem they present is susceptible we think of a rational solution. Though their brains are of fair and respectable size, they are, in their qualities and efficiency, a very inferior branch of the American Race. Of all the higher qualities of that Race they appear to be destitute. "They are commonly," says our author, on the authority of Lewis and Clark, "of diminutive stature, badly shaped, and their appearance by no means prepossessing. They have broad, thick, flat feet, thick ancles, and crooked legs." * * * * "The legs of the females particularly are ill shaped and swollen." This unsightly condition of the legs and ancles is attributed by some (though we think erroneously) to "tight bandages of beads and strings, worn round the ancles by the women, which prevent the circulation of

4*

the blood," and thus produce the preternatural bulk and clumsiness of the limbs. This cannot be received as correct physiology. The blood nourishes and vitalizes the organs which it visits. Let its circulation in them be "prevented," and they will become *smaller* instead of larger, for want of nourishment; or disease will invade them, and run into ulceration, if not into gangrene. By the well known action of the bandage, when tight, this is conclusively proved. To some other cause therefore must this thickness and shapeless clumsiness of the limbs be ascribed.

It is hardly we think to be doubted, that the general inferiority of the "Flat-head" Indians is attributable to the injury inflicted by compression on the brain. Were that compression made on the stomach, heart or lungs, no one will doubt that serious mischief would be the certain issue. Hence the well-known disasters of the practice of corsetting, in the production of dyspepsia, pulmonary consumption, disease of the heart, and other grievous and often fatal affections.

But the brain is an organ equal at least in standing and importance to any of the thoracic or abdominal viscera. We have pronounced it in many respects superior, and still maintain the same belief. Through the medium of the spinal cord and nerves, its influence is transmitted with great and essential effect, to every portion, we might say, directly or indirectly, to every fibre of the system. That it therefore can be compressed and distorted, if not literally *dislocated*, with greater impunity than other organs in no respect its superior, but decidedly the reverse, is exceedingly improbable—not to employ a stronger term, and say *impossible*. The "Flat-heads" are palpably of the same race with the great body of the North American Indians east of the Rocky Mountains. They are also fed as plentifully, clothed as well, and lodged as comfortably, and occupy a climate as pleasant and salubrious. Still are they comparatively of a degenerate caste. For their personal inferiority then to the other tribes of their family, no adequate cause presents itself *from without.* Yet some general and powerful cause of inferiority in them exists. That that cause is *internal* therefore, there is no ground to deny, and but little, in our estimation, to doubt. And we perceive nothing more likely to produce the mischief, than the violence done indirectly to the whole system, by the compression of the brain. And that no deterioration of intellect results from such compression, it requires more credulity than we possess to believe, or deem possible. In our opinion, a more incredible position can hardly be put or even imagined. The process is but little less in character than an infantile *chronic apoplexy*—or a fracture and long-continued depression of the skull. The marvel is, not that such violence done to the brain should seriously derange the functions of that viscus; but that it does not produce fatuity or death. The following are the dimensions of the cranium of a "Flat-head" of the largest size.

"PLATE XLV.—KILLEMOOK.

"*Measurements.*

| | | |
|---|---|---|
| "Longitudinal diameter, - - - | 6.9 | inches. |
| Parietal diameter, - - - | 6.3 | " |
| Frontal diameter, - - - | 4.9 | " |
| Vertical diameter, - - - | 4.8 | " |
| Inter-mastoid arch, - - - | 15.7 | " |
| Inter-mastoid line, - - - | 4 | " |
| Occipito-frontal arch, - - - | 14 | " |
| Horizontal periphery, - - - | 21 | " |
| Extreme length of the head and face, | 8.5 | " |
| Internal capacity, - - - | 92 | cubic inches. |
| Capacity of the anterior chamber, - | 34 | " " |

| | | |
|---|---|---|
| Capacity of the posterior chamber, | 58 | " " |
| Capacity of the coronal region, - | 19.3 | " " |
| Facial angle, - - - - | 73 | degrees." |

This is the largest but two of all the posterior chambers of the skull, that Dr. Morton has measured. It testifies of course to the inordinate animality in the savage who possessed it. But on these points, full of interest and instruction as they are, we can dwell no longer, but must hasten toward the close of our review.

Having terminated his measurements and calculations, after toiling through a degree of industry, patience, and labor that, without exaggeration, may be pronounced stupendous, Dr. Morton thus expresses himself, in the language of one who is emboldened by a consciousness of having faithfully endeavored to acquit himself of his duty.

"In conclusion, the author is of the opinion that the facts contained in this work tend to sustain the following propositions:

"1st. That the American Race differs essentially from all others, not excepting the Mongolian; nor do the feeble analogies of language, and the more obvious ones in civil and religious institutions and the arts, denote any thing beyond casual or colonial communication with the Asiatic nations; and even these analogies may perhaps be accounted for, as Humboldt has suggested, in the mere coincidence arising from similar wants and impulses in nations inhabiting similar latitudes.

"2d. That the American nations, excepting the Polar tribes, are of one Race and one species, but of two great Families, which resemble each other in physical, but differ in intellectual character.

"3d. That the cranial remains discovered in the mounds, from Peru to Wisconsin, belong to the same race, and probably to the Toltecan family."

Having laid down these propositions, in the form of corollaries deduced from the body of the work, our author offers, on the comparative size of the brains of the five races of men of whom he had treated, the following interesting and important observations. The facts they embrace are the result of admeasurements; and, as far as they extend, they put at rest the question of the relative magnitude of the Caucasian brain. We feel persuaded that, as soon as they shall be made known to him, even Tiedemann himself and his stubborn adherents, hostile as they are to the doctrines of phrenology, will cease to contend that the brain of the African is equal in size to that of the Caucasian. With equal truth may they contend for identity in the colour of the skin, the figure of the nose, and the entire character of the lips and hair of the two Races. Never were the blindness and deceptiveness of professional prejudice more doggedly manifested. The following are the observations to which we allude.

NOTE.—*On the Internal capacity of the Cranium in the different Races of men.*—Having subjected the skulls in my possession, and such also as I could obtain from my friends, to the internal capacity measurement already described, I have obtained the following results. The mean of the American Race (omitting a fraction) is repeated here merely to complete the table. The skulls of idiots and of persons under age were of course rejected.

| "Races. | No of Skulls. | Mean internal capacity in cubic inches. | Largest in the series. | Smallest in do. |
|---|---|---|---|---|
| Caucasian, | 52 | 87 | 109 | 75 |
| Mongolian, | 10 | 83 | 93 | 69 |
| Malay, | 18 | 81 | 89 | 64 |
| American, | 147 | 80 | 100 | 60 |
| Ethiopian, | 29 | 78 | 94 | 65 |

"1. The Caucasians were, with a single exception, derived from the lowest and least educated class of society. It is proper however to mention that but three Hindoos are admitted in the whole number, because the skulls of these people are probably smaller than those of any other existing nation. For example seventeen Hindoo heads give a mean of but *seventy-five* cubic inches."

After a few farther remarks on the Caucasians, Mongolians, Malays, and Ethiopians, which it is not important for us to quote, Dr. Morton subjoins:

"5. Respecting the American Race, I have nothing to add, excepting the striking fact, that, of all the American nations the Peruvians had the smallest heads, while those of the Mexicans were something larger, and those of the barbarous tribes the largest of all, viz:

| | | | |
|---|---|---|---|
| "Toltecan nations. | Peruvians collectively, | 76 | cubic inches. |
| | Mexicans collectively, | 79 | " |
| Barbarous tribes as per table, | | 82 | " |

"An interesting question remains to be solved, viz: the relative proportion of brain in the anterior and posterior chambers of the skull in the different races; an inquiry for which I have hitherto possessed neither sufficient leisure, nor adequate materials."

As connected with this question, and tending toward a solution of it, the following statement is worthy of notice.

On comparing with each other an American and a Caucasian brain, very nearly equal in size, and each being in form about an average of its race, the size of the three lobes was materially different.

In the Caucasian brain, the front lobe, which is the seat of intellect, was much larger than in the American, and its convolutions considerably bolder and more prominent.

In the American brain, the middle lobe, the seat of the animal and the semi-animal propensities, was in an equal degree larger than in the Caucasian, and its convolutions deeper.

And in the Caucasian brain, the posterior lobe, the seat of the domestic and social affections, was much larger, and more strongly marked in its convolutions than in the American.

These facts are in beautiful correspondence with the well-known differences in the characters of the two races. The Caucasian is superior, according to the indication of his brain, in the intellectual, social, and domestic faculties, and the American in the animal and semi-animal. C. C.

